# A Case of Atypical Lymphocytic Lobular Panniculitis

**DOI:** 10.7759/cureus.7134

**Published:** 2020-02-28

**Authors:** Catherine S Ni, G. Peter Sarantopoulos, Melvin Chiu

**Affiliations:** 1 Dermatology, David Geffen School of Medicine at University of California Los Angeles, Los Angeles, USA; 2 Pathology, David Geffen School of Medicine at University of California Los Angeles, Los Angeles, USA; 3 Dermatology, Keck School of Medicine at the University of Southern California, Los Angeles, USA

**Keywords:** panniculitis, dermatopathology, lobular panniculitis, atypical lymphocytic

## Abstract

Atypical lymphocytic lobular panniculitis (ALLP) is a rare T-cell dyscrasia of the subcutaneous fat. It typically presents with indurated erythematous nodules on the lower extremities and often will have a relapsing and remitting course. The cause is unknown, but clinically and histopathologically it shares similarities to lupus panniculitis (LP) and subcutaneous panniculitis-like T-cell lymphoma (SPTCL). It generally has an indolent course, and may best be treated like indolent versions of SPTCL with systemic steroids and immunosuppressive medications.

## Introduction

Atypical lymphocytic lobular panniculitis (ALLP) is a rare T-cell dyscrasia of subcutaneous fat initially described as indeterminate lymphocytic lobular panniculitis by Magro et al. in 2001 and later redesignated as ALLP [1-2]. The clinical presentation is similar to other panniculitides with indurated erythematous nodules, often on the lower extremities with a relapsing and remitting course. The cause of ALLP is unknown. In this case report, we describe a patient with ALLP who had temporary improvement with courses of prednisone and intralesional triamcinolone acetonide injections, no improvement with hydroxychloroquine, and incidental discovery and resection of a benign meningioma without resolution of her ALLP.

## Case presentation

**Figure 1:**
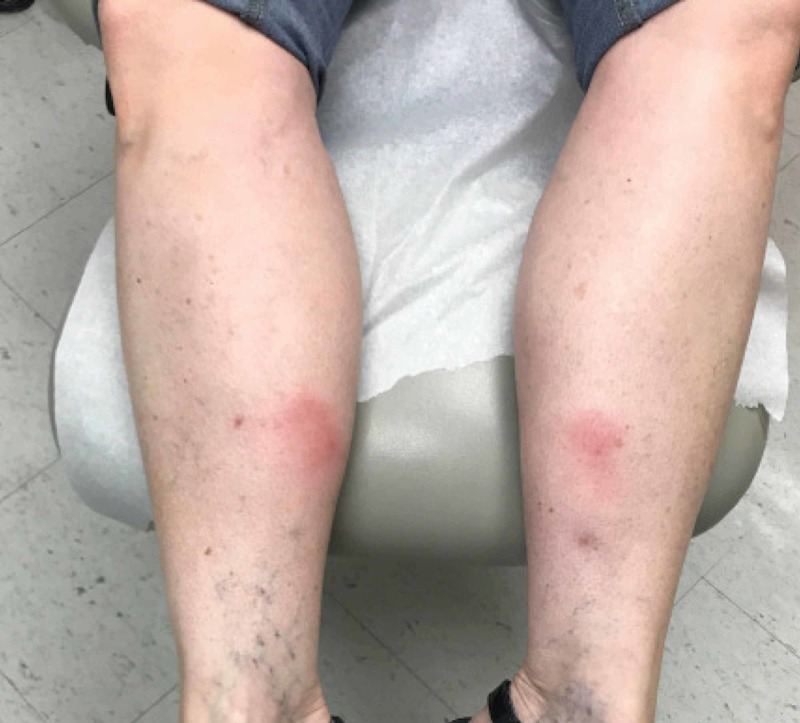
Erythematous nodules on bilateral lower extremities

A 46-year-old female presented to the ambulatory dermatology clinic with a nine-year history of recurrent tender red nodules on bilateral legs and occasionally her thighs. The lesions would sometimes regress spontaneously, but would frequently recur at a nearby site. She did not note any triggers or remitting factors, and the lesions would not ulcerate. She was otherwise in good health and not on any medications. Her past medical history was only significant for a history of non-melanoma skin cancers excised in the past. She did not have any fevers, chills, night sweats, or constitutional symptoms, and she did not note any unintended weight loss. She also denied symptoms of chronic cough, headaches, weakness, or seizures. Physical examination revealed tender indurated red nodules on bilateral anterior and posterior aspects of her legs without overlying scale or ulceration (Figure 1).

**Figure 2:**
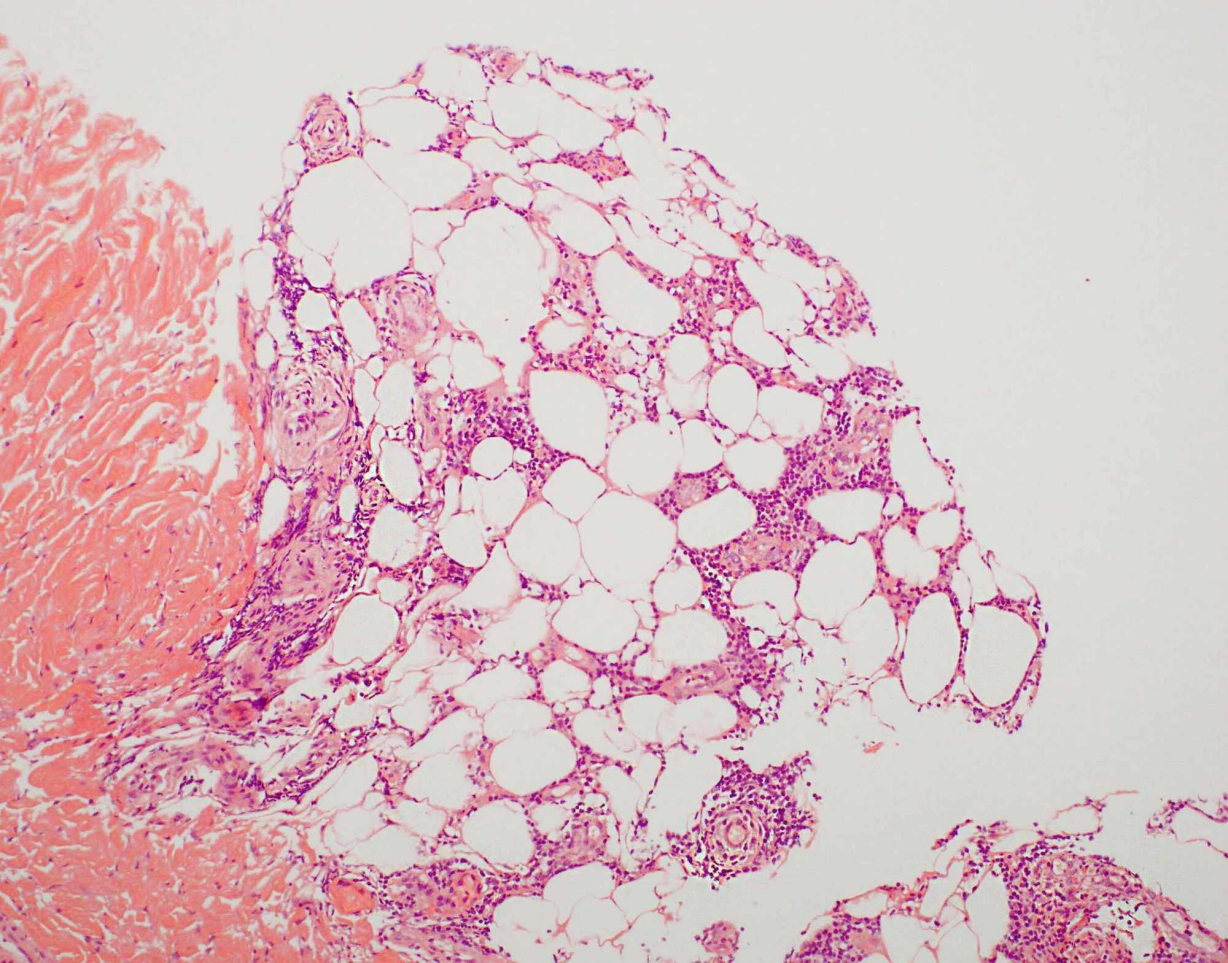
Photomicrograph, hematoxylin and eosin stain, 100x magnification Atypical lymphoid infiltrate surrounding the subcutaneous fat lobules.

**Figure 3:**
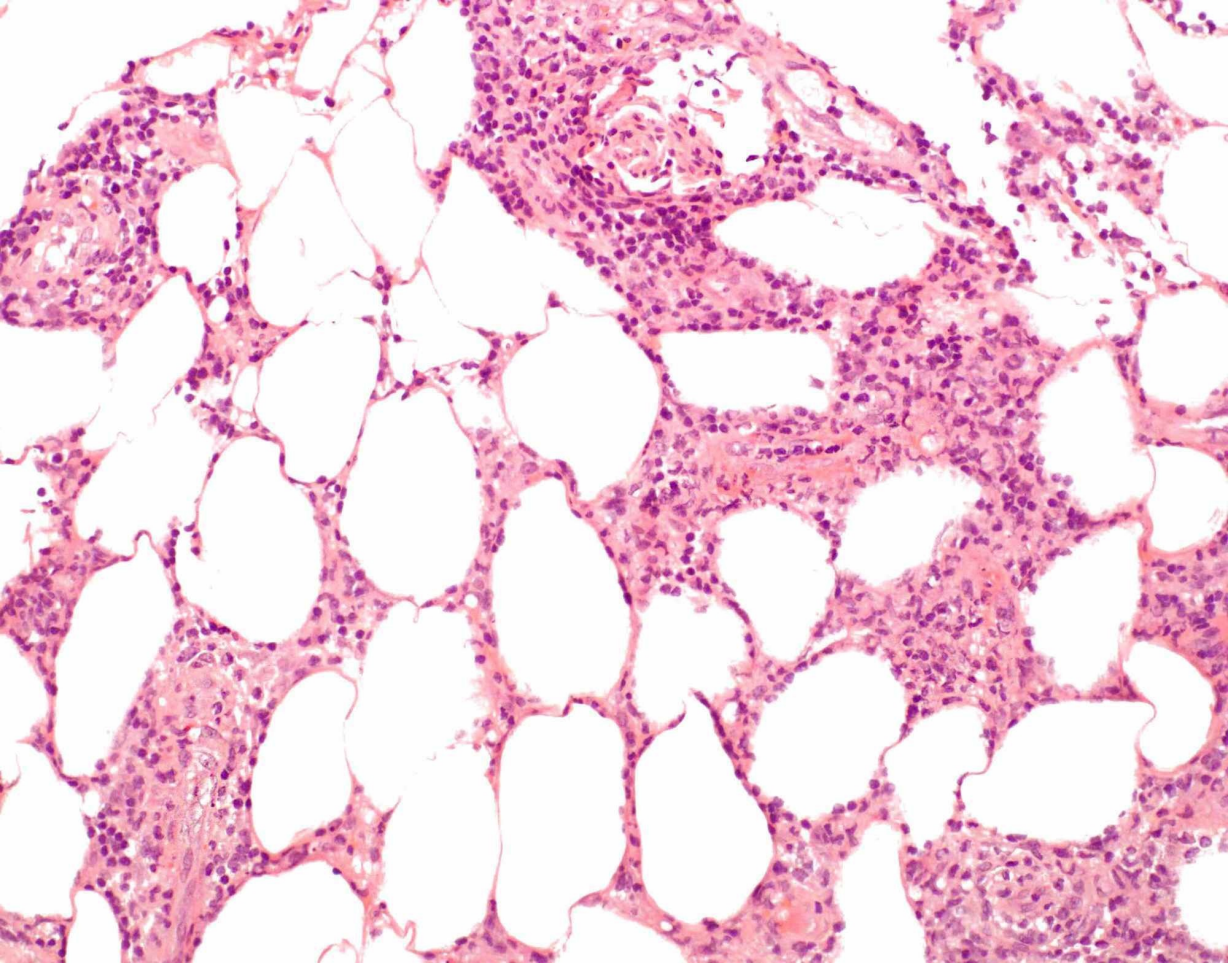
Photomicrograph, hematoxylin and eosin stain, 200x magnification Atypical lymphocytes surrounding the subcutaneous fat lobules.

**Figure 4:**
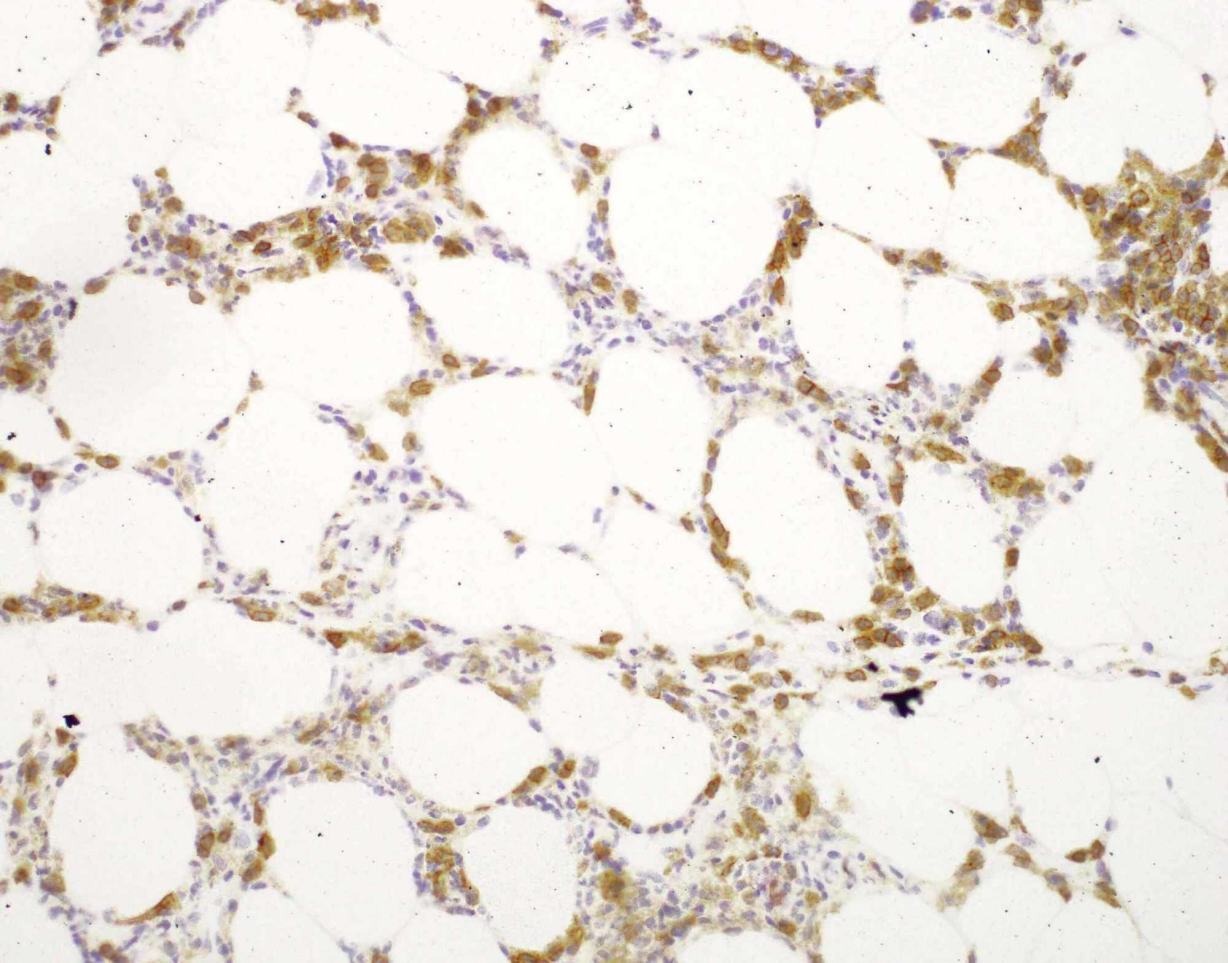
Photomicrograph, immunohistochemistry of CD4+ cells, 200x magnification The lymphocytic infiltrate is predominantly CD4+.

**Figure 5:**
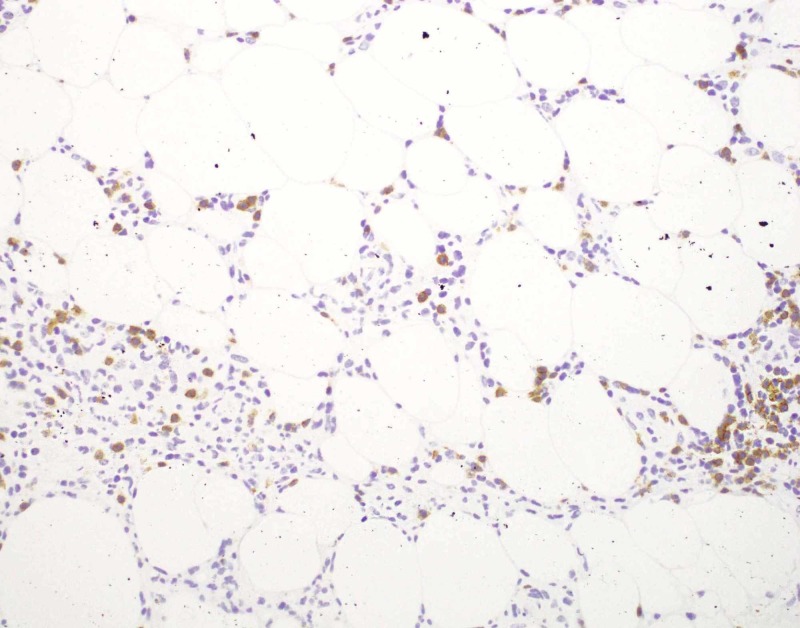
Photomicrograph, immunohistochemistry for CD8, 200x magnification A minority of the cells in the lymphoid infiltrate were CD8+.

**Figure 6:**
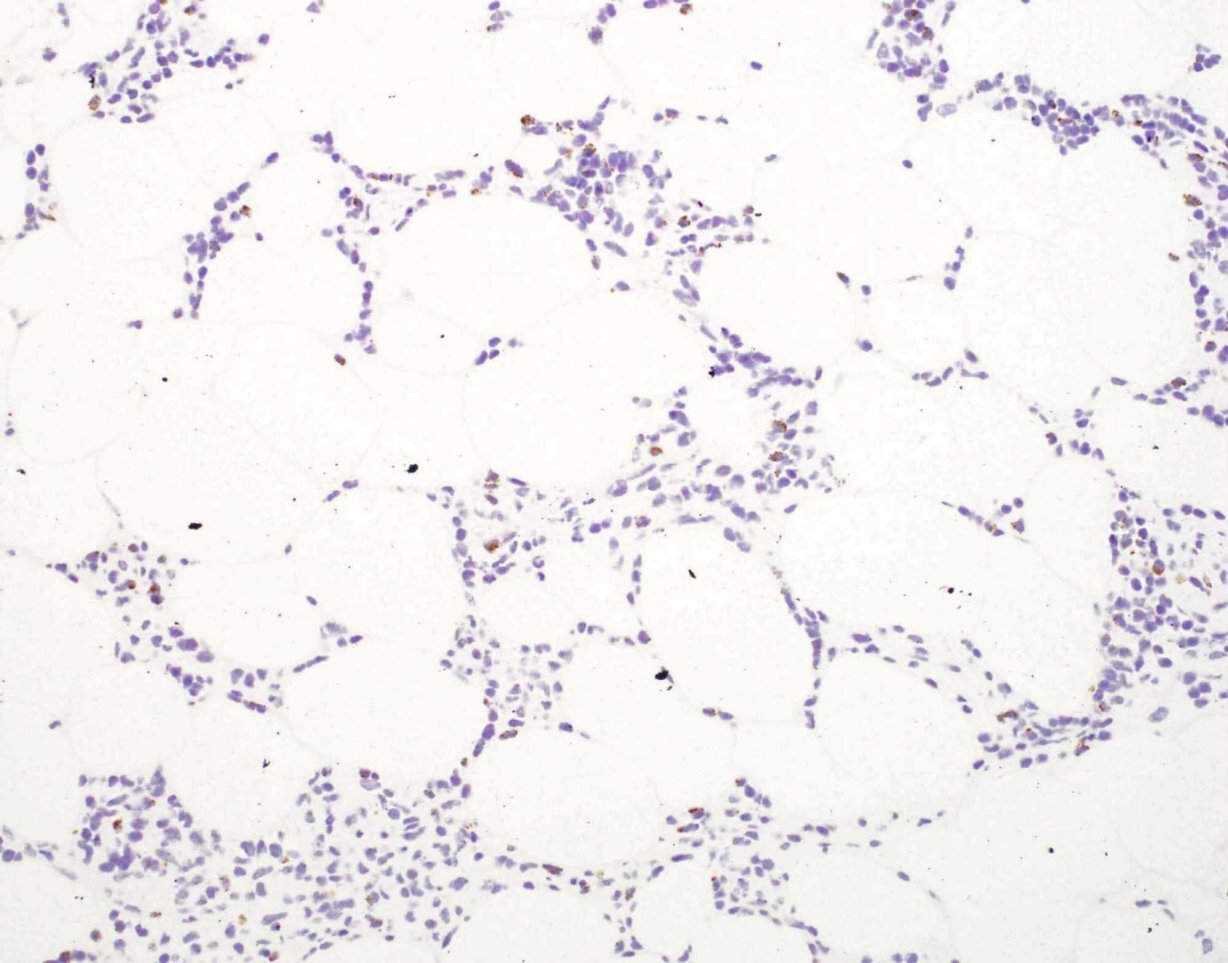
Photomicrograph, immunohistochemistry for granzyme, 200x magnification The cells in the lymphocytic infiltrate are predominantly granzyme negative.

The patient had several punch biopsies taken over the course of three years. Previous punch biopsies demonstrated a mildly to moderately dense infiltrate of small to medium sized lymphocytes predominantly around fat lobules with little to no atypia and variable rimming of lymphocytes around fat lobules. Another punch biopsy was obtained and findings were similar to previous biopsies. Histopathology showed an atypical lymphoid infiltrate in a predominately lobular pattern (Figures 2-3). Immunohistochemistry showed T cells were primarily CD4+ with some CD8+ T cells (Figures 4-5). Scattered cells stained positive for T-cell intracellular antigen-1 (TIA-1), but granzyme was negative (Figure 6). There was no significant rimming of adipocytes with atypical lymphocytes. T cell gene rearrangement studies were positive for clonal T cells in one sample, but negative or indeterminate in others. Laboratory testing showed normal complete blood count and comprehensive metabolic panel, negative tuberculosis interferon gamma release assay, normal lactase dehydrogenase, normal serum protein electrophoresis, normal serum immunoglobulin levels, normal serum lipase and amylase levels, negative anti-nuclear antibody titer, and a normal alpha 1 antitrypsin genotype of Pi*MM.

This constellation of clinical, histologic, and laboratory findings were consistent with a diagnosis of atypical lymphocytic lobular panniculitis. The patient received short courses of prednisone and intralesional triamcinolone acetonide injections with temporary relief. Empiric trials of supersaturated potassium iodide and colchicine were not effective and not tolerated by the patient due to gastrointestinal side effects. The patient was started on hydroxychloroquine 200 mg once daily (3 mg/kg/d) for three months and underwent radiographic imaging. The patient had a normal computed tomography (CT) scan of her chest, abdomen, and pelvis. A full body positron emission tomography (PET)/CT scan and brain magnetic resonance imaging (MRI) detected an incidental benign mass in the left vertex of her brain that was excised and confirmed to be a benign meningioma. After resection of her meningioma, she discontinued her hydroxychloroquine and continued to experience episodes of ALLP with only intermittent intralesional triamcinolone acetonide injections to treat her ALLP recurrences.

## Discussion

ALLP is a recently described entity that is thought to lie on a spectrum of cutaneous T-cell lymphoid dyscrasias--with lupus panniculitis (LP) on one end and subcutaneous panniculitis-like T-cell lymphoma (SPTCL) on the other [1-3]. In 2001, Magro et al. first described a subset of patients with indeterminant lymphocytic lobular panniculitis (ILLP) who exhibited some features of both LP and SPTCL, yet lacked diagnostic features of either [1]. In 2004, this was subsequently re-named “atypical lymphocytic lobular panniculitis.” Magro et al. described a series of twelve patients with ALLP who had waxing and waning bruise-like plaques and lacked any clinical or laboratory stigmata of lupus erythematosus or SPTCL, the latter of which can display a clinically aggressive course [2].

The clinical differential diagnosis of ALLP includes erythema nodosum, erythema induratum, nodular vasculitis, LP, pancreatic panniculitis, and SPTCL. Biopsy can often distinguish between these entities. Erythema nodosum presents primarily as a septal panniculitis; whereas, the others are primarily lobular panniculitides. Erythema induratum (or nodular vasculitis when not associated with tuberculosis) is usually associated with tuberculosis and will often show lobular or mixed septal and lobular panniculitis with prominent vasculitis on histopathology. Pancreatic panniculitis is usually identified by subcutaneous fat necrosis.

Distinguishing between LP, ALLP, and SPTCL can be more difficult. All three entities favor the proximal extremities, buttocks, and trunk. LP and ALLP tend to have waxing and waning courses; whereas, SPTCL tends to be persistent and progressive. LP occurs in 1%-3% of patients with cutaneous lupus erythematosus [4]. SPTCL patients may also exhibit fever, other constitutional symptoms, and hemophagocytic syndrome [5].

These three entities are difficult to distinguish histologically as well. ALLP and SPTCL both exhibit an infiltrate of atypical lymphocytes in the dermis in an epidermotropic and eccrinotropic pattern, as well as plump rounded histiocytes in the interstitial reticular dermis. SPTCL exhibits a greater degree of lymphoid atypia, more extensive fat necrosis, and rimming of adipocytes by neoplastic T cells. Prominent erythrocyte phagocytosis by histiocytes and angiodestructive changes with luminal thrombosis are seen in SPTCL but not in ALLP [3]. Phenotypically, ALLP and SPTCL both show variable reduction in pan T-cell markers-CD3, CD5, and CD7. While ALLP may also exhibit a reduced CD4:CD8 ratio, SPTCL often shows a predominance of CD8 lymphocytes that are of the cytotoxic subtype, as evidenced by the expression of perforin and T-cell intracellular antigen-1 (TIA-1) [2].

Clonal restriction is observed in both ALLP and SPTCL but not LP. Distinguishing characteristics of LP include prominent lymphoid germinal centers, hyalinosis of the fat lobule, prominent dermal and subcuticular mucin deposition, an interface dermatitis, and a positive lupus band on direct immunofluorescence [3].

As a cutaneous lymphoma, SPTCL is included in the 2018 World Health Organization-European Organization for Research and Treatment of Cancer (WHO-EORTC) classification of primary cutaneous lymphomas. ALLP, however, is not technically a lymphoma and not listed in the 2018 WHO-EORTC cutaneous lymphoma categorization system [6].

The cause of ALLP is currently unknown, and a review of the literature failed to identify reports of ALLP associated with benign or malignant tumors. Purported triggers of ALLP in one case series include sinusitis, recurrent Streptococcal pharyngitis, and upper respiratory infections in four patients, but no identifiable trigger in the remaining eight patients. One patient had a remote history of non-Hodgkin’s lymphoma, but the other patients reported did not have a history of malignancy [2]. Our patient did not report any triggers, and we believe the patient's meningioma was merely incidental, as resection of the tumor did not lead to resolution of her ALLP.

Both LP and ALLP may respond to prednisone and hydroxychloroquine. There are reports of ALLP responding to systemic retinoids, alemtuzumab (an anti-CD52 monoclonal antibody), and non-steroidal inflammatory drugs as well [2]. Patients with SPTCL are often treated with combination chemotherapy such as cyclophosphamide, doxorubicin, vincristine, and prednisone (CHOP) or CHOP-like courses, sometimes in combination with alemtuzumab or followed by stem cell transplant [7]. However, more recent studies suggest that patients with the less aggressive SPTCL alpha-beta phenotype without hemophagocytic syndrome have a good prognosis and should be treated with systemic immunosuppression such as systemic steroids, methotrexate, or cyclosporine instead of multi-agent chemotherapy [8-9].

## Conclusions

In conclusion, ALLP represents a rare T-cell dyscrasia of the subcutaneous fat with a chronic and relapsing course. The cause of ALLP is currently not known, but It shares clinical and histopathologic characteristics with LP and SPTCL and differentiation can often be difficult. ALLP’s indolent course may display similarities to the less aggressive variant of SPTCL, and similarly, therapy with systemic steroids and immunomodulatory or immunosuppressive medications such as hydroxychloroquine, methotrexate, and cyclosporine may be appropriate.
